# Diagnostic journey for individuals with fibrous dysplasia / McCune albright syndrome (FD/MAS)

**DOI:** 10.1186/s13023-024-03036-w

**Published:** 2024-02-07

**Authors:** Kaiyang Song, Roshi Shrestha, Heather Delaney, Rohit Vijjhalwar, Alison Turner, Maria Sanchez, Muhammad Kassim Javaid

**Affiliations:** 1 Medical Sciences Division, University of Oxford, Headley Way, OX3 9DU Oxford, USA; 2 NDORMS, University of Oxford, Oxford, USA; 3 Patient representative, Oxford, USA

**Keywords:** Fibrous dysplasia, McCune -Albright Syndrome, Diagnosis, Epidemiology, Online

## Abstract

**Background:**

Reducing delayed diagnosis is a significant healthcare priority for individuals with rare diseases. Fibrous Dysplasia/ McCune Albright Syndrome (FD/MAS) is a rare bone disease caused by somatic activation mutations of *NASA.* FD/MAS has a broad clinical phenotype reflecting variable involvement of bone, endocrine and other tissues, distribution and severity. The variable phenotype is likely to prolong the diagnostic journey for patients further.

**Aim:**

To describe the time from symptom onset to final diagnosis in individuals living with FDMAS.

**Methods:**

We used the UK-based RUDY research database (www.rudystudy.org), where patients self-report their diagnosis of FD/MAS. Participants are invited to complete the diagnostic journey based on the EPIRARE criteria.

**Results:**

51 individuals diagnosed with FD/MAS were included in this analysis. Among them, 70% were female, and the median age was 51.0 years (IQR 34.5–57.5]. 12 (35%) individuals reported McCune Albright Syndrome, 11 (21.6%) craniofacial and 11(21.6%) for each of poly- and mono-ostotic FD and 6 (11.8%) did not know their type of FD/MAS. Pain was the commonest first symptom (58.8%), and 47.1% received another diagnosis before the diagnosis of FD/MAS. The median time to final diagnosis from the first symptom was two years with a wide IQR (1,18) and range (0–59 years). Only 12 (23.5%) of individuals were diagnosed within 12 months of their first symptoms. The type of FD/MAS was not associated with the reported time to diagnosis. Significant independent predictors of longer time to final diagnosis included older current age, younger age at first symptom and diagnosis after 2010.

**Conclusion:**

Individuals with FDMAS have a variable time to diagnosis that can span decades. This study highlights the need for further research on how to improve diagnostic pathways within Orthopaedic and Ear, Nose and Throat (ENT)/Maxillofacial services. Our data provides a baseline to assess the impact of novel NHS diagnostic networks on reducing the diagnostic odyssey.

**Supplementary Information:**

The online version contains supplementary material available at 10.1186/s13023-024-03036-w.

## Background

Fibrous Dysplasia / McCune Albright Syndrome (FD/MAS) is a rare disease that arises following somatic activation mutations of the *GNAS* gene, which codes for the α-subunit of the *G*_*s*_ stimulatory protein [1]. The widespread nature of *G*_*s*_*α* signalling across various tissues explains the involvement of bone, skin and endocrine organs. Even the skeletal presentations of FD/MAS are heterogenous, both in severity and location, affecting the craniofacial, axial and/or appendicular skeleton. Patients with FD/MAS often present with bone pain, joint deformities and are at a higher risk of developing stress and impending fractures. Whilst the majority of patients present with monostotic lesions, others present with a polyostotic phenotype, most commonly involving the femur, tibia and skull [2]. Moreover, endocrinopathies typically associated with FD/MAS include growth hormone excess [3], precocious puberty [4], Cushing syndrome [5] and renal phosphate wasting [6].

Although the prevalence of FD/MAS remains largely unknown, estimates vary between 1:100,000 and 1:1,000,000 [7]. It is widely documented that individuals with rare diseases, defined in the EU as affecting fewer than 1 in 2000 people in the general population [8, 9],frequently experience diagnostic delay, amongst other shortcomings in clinical care. In the UK, the Rare Diseases framework, states that a priority in the management of these conditions is to avoid a “diagnostic odyssey” and reach a timely final diagnosis [8]. A survey of individuals living with rare diseases highlighted that the commonest challenge experienced was receiving the correct diagnosis (30% of responses), above healthcare professionals’ awareness of rare diseases (19%) and access to appropriate medical care (17%) [8].

The diagnosis of FD/MAS is based on clinical suspicion, following an assessment of skeletal, endocrine, dermatological and soft tissue manifestations [10]. Common investigations include radiological and histological analysis of affected sites; in rare cases, genetic analysis is required to inform the clinical diagnosis [10].

Prompt diagnosis of the subtype of FD/MAS and any biochemical imbalances is crucial for improving patients’ symptoms and quality of life. For example, in patients with endocrinopathies, optimising phosphate balance and endocrine abnormalities can reduce the progression of skeletal disease. Moreover, regarding skeletal deformities, early review by orthopaedic surgeons facilitates the identification of patients with skeletal deformities that may benefit from prophylactic surgery to manage worsening pain, deformity and reduce patients’ fracture risk [10].

Nevertheless, the time from potential initial symptoms to final diagnosis in FD/MAS patients is yet to have been performed, despite cases of diagnostic delay being well-documented [11, 12]. Here, we analysed data collected from the Rare and Undiagnosed Disease Study (RUDY study), an ongoing UK-based prospective cohort study, to describe the diagnostic journey for adults with FD/MAS.

## Methods

### Study population

Data from the Rare and Undiagnosed Diseases Study (RUDY study) was used for this study. The RUDY study is an ongoing UK-based multi-centre prospective cohort study, launched in 2014, which aims to improve our understanding of the impact of rare diseases [13]. It is web-based where participants can securely complete forms and questionnaires at 6 monthly intervals (Sup. Figure 1). Participants give online dynamic consent for their data to be collected and used for research. RUDY is accessed through www.rudystudy.org and promoted to patients with FD/MAS by the Fibrous Dysplasia Support Society UK, as well as social media. Only participants aged 16 years and over with a self-reported diagnosis of Fibrous Dysplasia or McCune-Albright syndrome were included. Our study used RUDY data collected between 2014 and 2022.

Ethics approval for the RUDY Study was given by the South Central Research Ethics committee after review (LREC 14/SC/0126 & RUDY LREC 17/SC/0501), and participants gave informed consent.

### Outcomes

The primary outcome was the time elapsed from the first potential symptom to the final diagnosis. Secondary outcomes were the time taken from the first General Practitioner (GP) visit and from the first hospital visit to the final diagnosis. One patient saw his GP in 2013 and received a final hospital diagnosis in 2013. However, their initial symptoms occurred in 1997 and they were seen in the hospital in the same year; this patient was excluded from the GP-to-hospital analysis.

### Predictors

Type of FD/MAS was grouped into four different groups: monostotic, polyostotic, cranio-facial and McCune Albright Syndrome. Some individuals had recorded more than one type of FDs. In this case, the hierarchy of the FD type (monostotic < polyostotic < cranio-facial < McCune Albright Syndrome) was used. If the type of FD was missing it was recorded as “Not known”. The diagnostic journey was recorded by the participants in the RUDY platform as part of the diagnostic form based on the EPIRARE recommendations. Symptom descriptions were coded into eight different groups (pain, premature menarche, café au lait lesions, lump, fracture, deformity, vision and limp). The symptom types were segregated as pain and non-pain for analysis. Other suggested diagnoses that were provided by clinicians before the final diagnosis of FD/MAS were divided into “Cancer” or “Non-cancer”. Finally, we analysed the type of healthcare professional seen by the participant on their diagnostic journey.

### Data analysis

We visually assessed for normality of variables. Patient characteristics and disease-related factors were summarised using descriptive statistics. Mean values were utilised for normally distributed data, while median values were employed when the data was not normally distributed. We compared diagnostic times before and after the publication of the Best Practice Guideline for FD/MAS in 2019 [10] and examined the for differences in participants with adult-onset symptoms vs. childhood onset with univariate and multivariable linear regression models. A level of significance of *p* < 0.05 was used. Diagnostic regression testing for residuals were used for the primary and secondary outcomes using RStudio (Version 2022.07.2).

## Results

One hundred sixty-five individuals that took part in RUDY study self-reported a diagnosis of FD/MAS. Out of these 165 participants, 114 in total were excluded (112 were missing the first symptom date and two were missing the final diagnosis date). Of the 51 individuals included, the median age was 51.0 [Interquartile range (IQR): 34.5–57.5] and 36/51 (70.6%) were female. Moreover, there were at least 10 patients in each FD/MAS type (Table 1).

**Table 1:** Patients and disease-related factors table

	Total (*N* = 165)	Included (*N* = 51)	Excluded (*N* = 114)	
**Age (years)**				
Median [Interquartile range (IQR)]	44.0 [32.0, 56.0]	51.0 [34.5, 57.5]	41.5 [30.2, 56]	
**Range** **Gender**				
Female	118 (71.5%)	36 (70.6%)	82 (71.9%)	
**Type of FD/MAS**				
Cranio-facial	14 (8.5%)	11 (21.6%)	3 (2.6%)	
McCune Albright Syndrome	22 (13.3%)	12 (23.5%)	10 (8.8%)	
Monostotic	27 (16.4%)	11 (21.6%)	16 (14.0%)	
Polyostotic	23 (13.9%)	11 (21.6%)	12 (10.5%)	
Not known	79 (47.9%)	6 (11.8%)	73 (64%)	
**First symptoms**				
Non pain	27 (16.4%)	21 (41.2%)	6 (5.3%)	
Pain	35 (21.2%)	30 (58.8%)	5 (4.4%)	
Missing	104 (63.0%)	0 (0%)	103 (90.4%)	
**Other diagnosis**				
None given	131(79.4%)	27 (52.9%)	104 (91.2%)	
Cancer	14 (8.5%)	10 (19.6%)	4 (3.5%)	
Non cancer	20 (12.1%)	14 (27.5%)	6 (5.3%)	

Overall, pain related first symptoms were most common (59%). Twenty four (47%) of individuals received another diagnosis before their final diagnosis, with 42% (10/24) of these patients receiving a cancer-related diagnosis. The median year of diagnosis was 2010 (IQR 1992, 2015). The median age of first symptoms and final diagnosis by type of FD/MAS is shown in Table 2.

**Table 2:** Age of potential first symptom and final diagnosis by type of FD/MAS

Type of FD	Median age at first symptoms [IQR]			Median age at final diagnosis [IQR]	
Cranio-facial	19 [12, 24]			28 [18, 40]	
McCune Albright Syndrome	5 [3, 9]			11 [6, 14]	
Monostotic	31 [14, 36]			37 [24, 45]	
Polyostotic	12 [9, 22]			29 [16, 40]	
Not known	13 [9, 26]			44 [33, 50]	
*P* value	0.12			*0.005*	

**Fig. 1:**
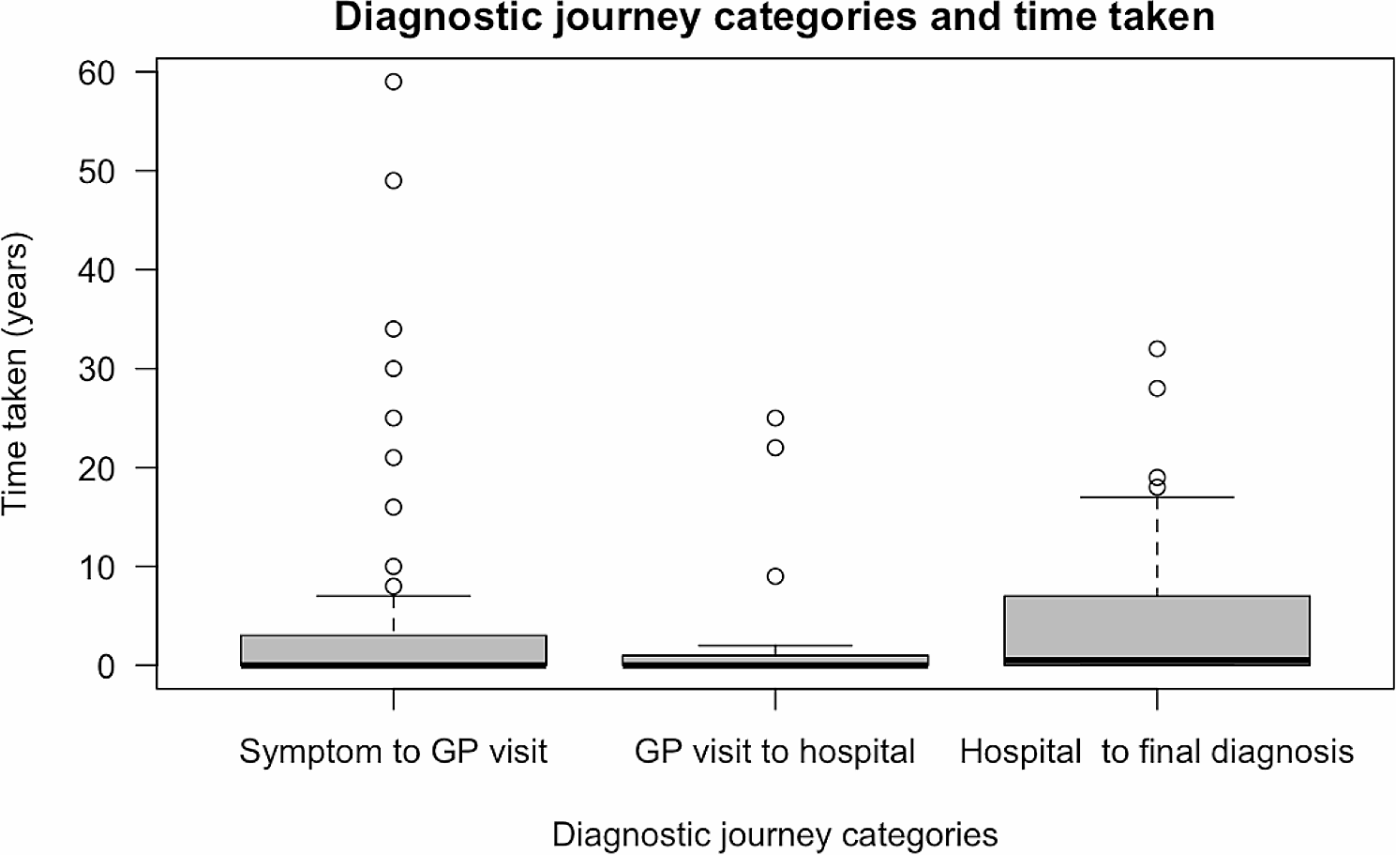
Time from first symptom onset to first GP, first hospital visit and final diagnosis Legend: the number of participants varied by group as not all patients saw a GP and were seen in a local hospital: symptom to GP visit (n=46); GP visit to hospital (n=45); Hospital to final diagnosis (n=51)

**Fig. 2:**
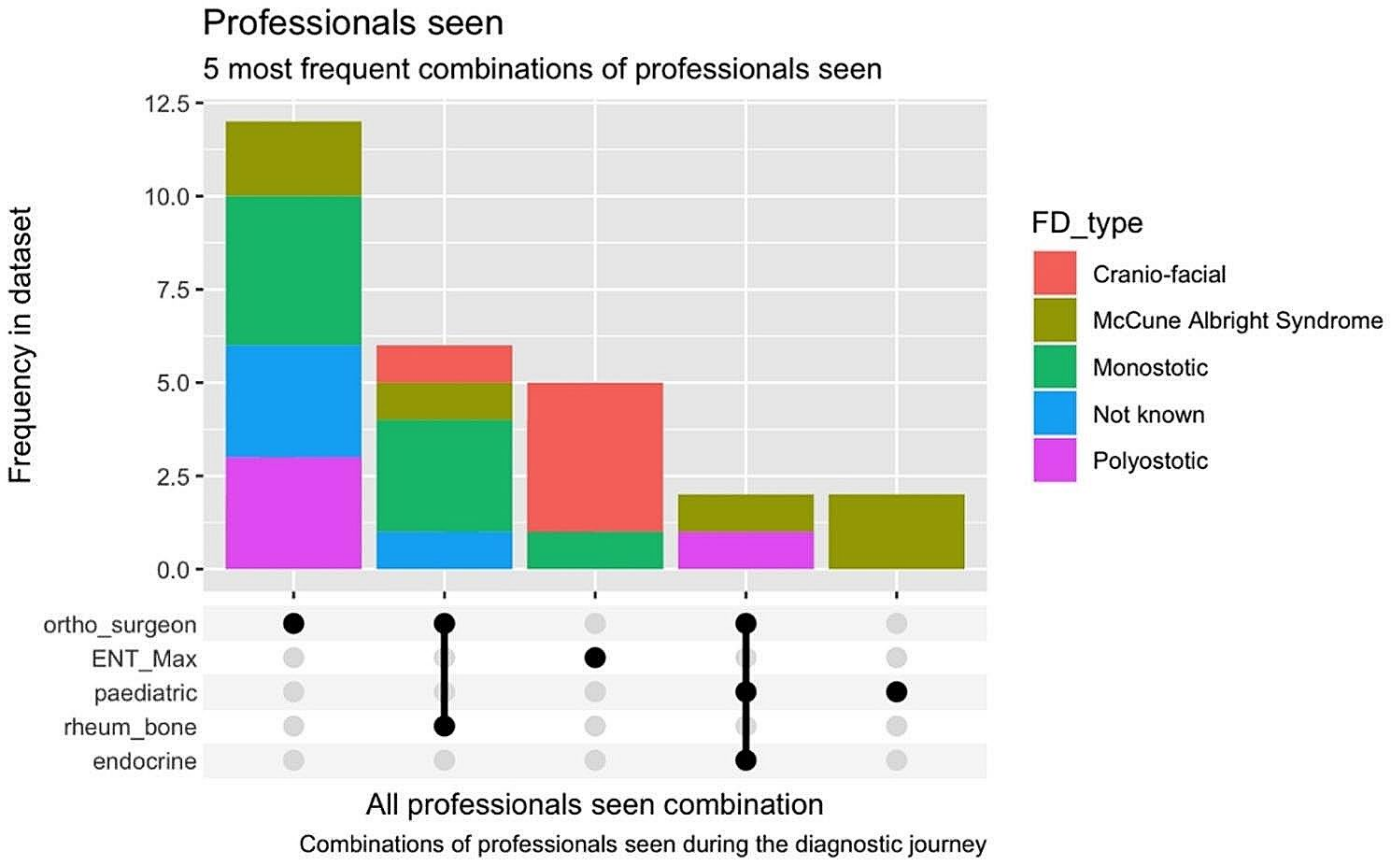
The 5 most frequent combinations of medical professionals seen by patients with different subtypes of FD/MAS during their diagnostic journey

Time from first symptom to final diagnosis ranged from 0 years to 59 years and the median years to diagnosis was 2[IQR 1,18] years, with 29 (56.9%) waiting more than 1 year. Of the 51 adults with first symptom to final diagnosis data, 46 (90.2%) reported seeing a GP first. The time between first symptom, GP appointment, hospital appointment and final diagnosis is shown in Fig. 1, with the longest time taken between symptom onset and GP visit and first hospital to final diagnosis (*p* = 0.08). The most common combination of clinicians seen prior to diagnosis is shown in Fig. 2. Across all 51 patients, the three predominant combinations of medical professionals seen were: those who exclusively consulted orthopaedic surgeons, those who consulted both orthopaedic surgeons and rheumatologists, and those who exclusively consulted Ear, Nose and Throat (ENT)/Oral and Maxillofacial surgeons. Individuals with monostotic and polyostotic FD predominantly consulted orthopaedic surgeons, while patients with craniofacial FD most frequently consulted ENT/Oral and Maxillofacial surgeons (Fig. 2).

**Fig. 3:**
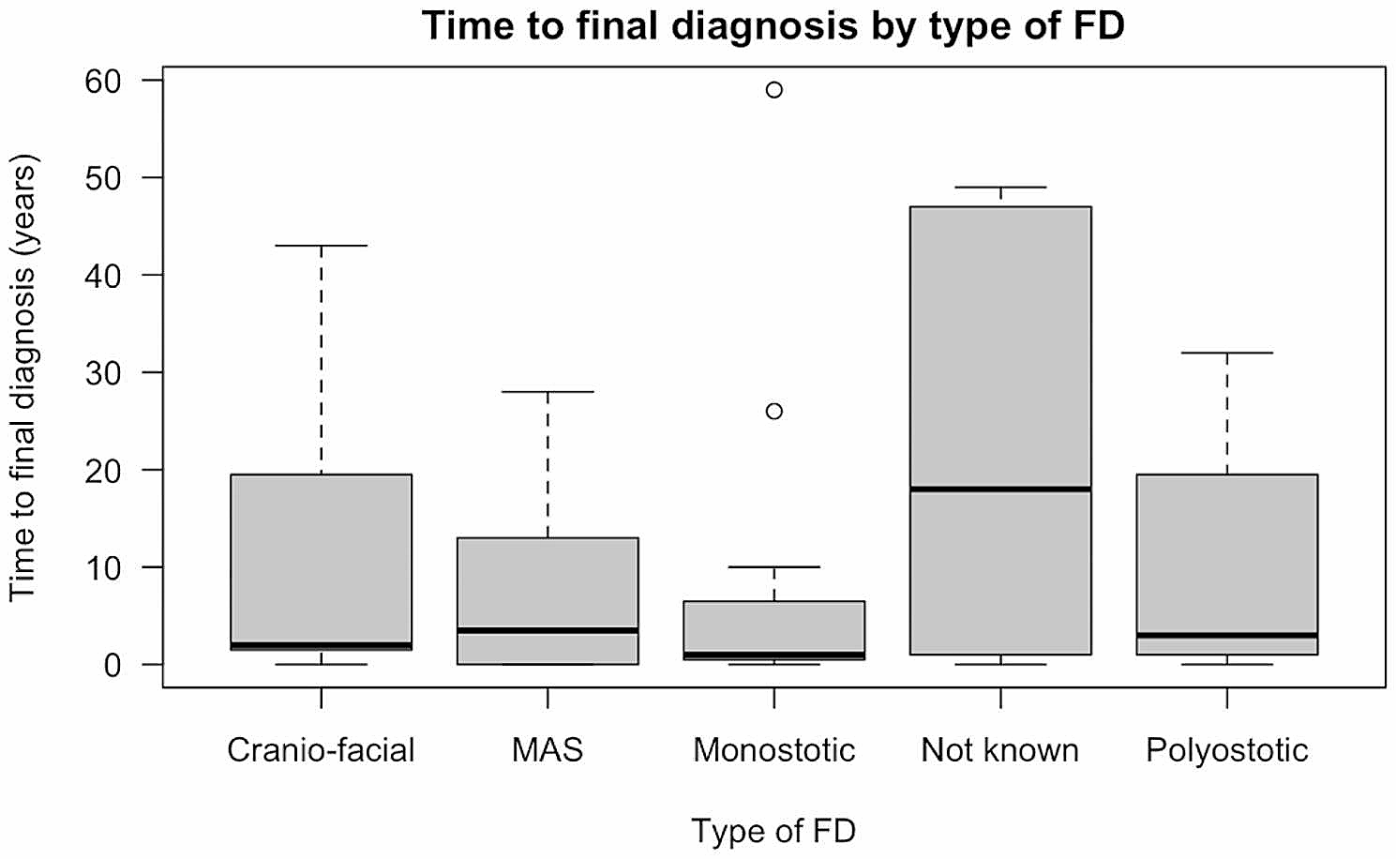
Time from first potential symptom to final diagnosis by type of FD/MAS

In the multivariate model, both older participants and those who experienced their first symptoms at a younger age reported a significantly longer time to diagnosis (Sup. Table 1). There are no significant differences by type of FD/MAS or type of first symptom (Sup.Table 1, Fig. 3). When we restricted the sample to those with symptom onset in adulthood (*n* = 19), the diagnostic journey was significantly longer for women and those diagnosed after 2010 (Sup. Table 2).

## Discussion

Reducing diagnostic delay is a major healthcare priority, especially for individuals with a rare disease. In this study of adults living with FD/MAS, the median time from potential first symptom to final diagnosis was 2 years with diverse range of clinical specialties involved in the diagnostic odyssey. The longest delay was from first visit to secondary care to final diagnosis.

The median age of diagnosis was shown to vary across different disease subtypes. For instance, monostotic FD which tends to have a mild clinical presentation, presented at a median age of 37 years old. However, McCune Albright was diagnosed most promptly, with the median age of diagnosis of 11 years old. This is likely elucidated by the fact that McCune Albright is the most severe form of the disease and often presents with extra-osseous features [10]. Nevertheless, the time elapsed between the median age of initial symptom and diagnosis was 6 years, reinforcing that diagnostic delay is still commonplace [11], and the fact that McCune Albright has a variable clinical presentation.

The vision of the International Rare Diseases Research Consortium is for all known rare diseases to be diagnosed within a maximum of one year from the date which they come to medical attention [14]. In Europe, the 2007 EURORDIS patient group survey indicated 25% of patients waited between 5 and 30 years and 40% received another first diagnosis [15]. A study of all rare disease patients in Spain, demonstrated the diagnostic journey varied by type of rare disease with a shorter diagnostic journey within a year for rare musculoskeletal disorders [16]. However, this study did not distinguish between types of rare musculoskeletal disorders. In hypophosphatasia, the mean time taken from initial symptoms to final diagnosis was around 10 years for adults, highlighting the importance of taking a comprehensive medical history at initial presentation [17]. In sternocostoclavicular hyperostosis (SCCH), a chronic inflammation condition affecting the anterior chest wall, the mean diagnostic delay across 52 patients was 5.6 ± 5.9 years [18]. The authors highlighted that greater recognition of the clinical characteristics of SCCH was crucial to achieving a more timely diagnosis.

Multiple factors influence the diagnostic delay, including factors related to the patient, healthcare providers, the healthcare system, and the characteristics of the disease itself. The diagnostic intervals can be divided into the duration from first symptom onset, the period during which the patient appraisal was being conducted, the time of the first primary care appointment, the time taken for referral to secondary care, and ultimately, the final diagnosis [19, 20]. The long diagnostic interval we have observed probably reflects the heterogeneity in both symptoms and age of clinical presentation in FD/MAS. This study identified the time from first hospital visit to final diagnosis as the highest attributable delay, highlighting a critical bottleneck in the diagnostic odyssey to address within secondary care. Contributing factors may include non-specific symptoms presenting to secondary care, lack of clinical knowledge, unclear referral pathways to expert centres and limited diagnostic laboratory tests for FD/MAS. The detection of the *GNAS* p.R201 variants in blood circulating cell free DNA has been shown to be effective for diagnosis of FD/MAS for those with endocrinopathy or high skeletal burden score [21]. However, the diagnostic uncertainty is higher in those with monostotic or craniofacial lesions, where this test is less sensitive. In our study, the date of first presentation to the healthcare system was via primary care in all but one case, reflecting the healthcare system of the NHS. While primary care provider can contribute to the diagnostic odyssey with a lack of coordination, information sharing and guidance [22], time from GP visit to hospital referral had a smaller contribution to the diagnostic journey in this patient group.

The diagnosis of FD/MAS usually requires musculoskeletal radiologists with clinical experts in rare diseases [10]. A number of European initiatives, including the European Reference Networks (ERNs), have been resourced to help reduce the diagnostic journey by supporting expert multidisciplinary meetings to shorten the diagnostic odyssey [23]. However, the BREXIT referendum of 2016 and subsequent withdrawal agreement, has excluded UK based clinicians and researcher from active participation in the ERNs, to the detriment of patient care [24], given no equivalent network has since been funded in the UK. Although the publication of recent, evidence-based, best practice guidelines has provided greater clarity over the radiological, histological and extra-skeletal manifestations of FD/MAS [10], it is anticipated that patients with FD/MAS will continue to experience diagnostic delay. Indeed, the rarity of the disease, in conjunction with its diverse clinical manifestations and multi-system involvement (requiring multi-specialty care), mean that diagnostic delay is likely. The NHS in the UK is about to institute a Rare Disease Collaborative Network for adults with rare bone diseases that should link every district hospital in England, Wales and Scotland to an adult rare bone disease network. Comparing these findings for individuals diagnosed after the network is functional may demonstrate the value of the network. The observation of a longer diagnostic interval in women and older individuals is consistent with other common and rare conditions [25]. The reasons behind this diagnostic disparities is likely multi-dimensional. Contributary factors may be related to the perception of symptoms in older patients and gender based differences in health seeking behaviour. These finding highlight the need for healthcare professionals not to overlook symptoms based on patients age alone as some symptoms, such as bone pain are more prevalent in the older individual with FD/MAS [26]. Further work is needed to understand the short, medium and long term impacts from a longer diagnostic delay for the patient, family and healthcare system perspectives [14].

The strength of this study is the use of patient-reported data for the type and timing of first potential symptom; these data are often inconsistently recorded in NHS clinical records. Previous studies that have compared patient reported symptoms with those recorded in the medical records have demonstrated fewer symptoms in the medical record and also poor concordance when symptoms are reported [27]^,^ [28]. Another strength is representation across the different sub-types of FD/MAS.

However, there are notable limitations. Firstly, there is the uncertainty regarding whether the first reported symptom was clinically related to the FD/MAS. Moreover, our study relied on patients self-reporting their diagnosis of FD/MAS. Given the nature of the RUDY study methodology, it is feasible that surveys may have been disproportionately completed by individuals who have higher levels of digital literacy. More generally, it is difficult to establish how representitive our sample of FD/MAS 51 patients was, and we acknowledge that important information may have been lost with the 114 patients for whom we were missing key data. Furthermore, we do not have data regarding molecular diagnosis, particularly genetic testing for GNAS, and self-reported diagnoses’ were not verified against medical records. Nevertheless, in the NHS, there is no systematic molecular characterization of FD/MAS, including for patients with solitary lesions. Another limitation is that for patients with FD/MAS subtypes that were diagnosed at a later age, where the alternative diagnoses may be more common (e.g. cancer), their older age may have affected the type and likelihood of other diagnoses being given. In addition, we cannot exclude that some patients did have a true dual diagnosis of cancer and FD/MAS, nevertheless previous literature has shown that the cancer risk in FD/MAS patients is low [29]. Finally, in milder subtypes, it is possible that clinicians identified FD lesions incidentally whilst investigating another medical condition. Nevertheless, there is growing literature to suggest that many patients with FD experience diagnostic delay as their symptoms are not recognized to be caused by FD lesions [10, 12].

In conclusion, this study has characterised the diagnostic odyssey experienced by adults with FD/MAS and demonstrated significant delays that are more pronounced in older adults and patients who experience their first symptoms at a younger age. Amongst patients with adult-onset symptoms, there is greater diagnostic delay for women. Our findings highlight the need for further research on how to improve diagnostic pathways within orthopaedic and ENT/maxillofacial services. Overall, this study provides a basis for researchers, clinicians and patients societies to co-develop interventions to reduce the diagnostic delay in this rare disease.

### Electronic supplementary material

Supplementary Material 1

## Data Availability

Anonymized data from the Rare and Undiagnosed Diseases study is available by direct request using www.rudystudy.org site and accessing the researcher tab.
