# Supplementary material for: Diagnostic journey for individuals with fibrous dysplasia / McCune albright syndrome (FD/MAS)
Source: Orphanet J Rare Dis. 2024 Feb 7;19:50. doi: 10.1186/s13023-024-03036-w (PMC10851567; doi:10.1186/s13023-024-03036-w)
Supplement: Supplementary file 1 — Supplementary Material 1 [file 13023_2024_3036_MOESM1_ESM.docx]

**Supplementary figure 1: The RUDY study questionnaire distributed to patients in this study**


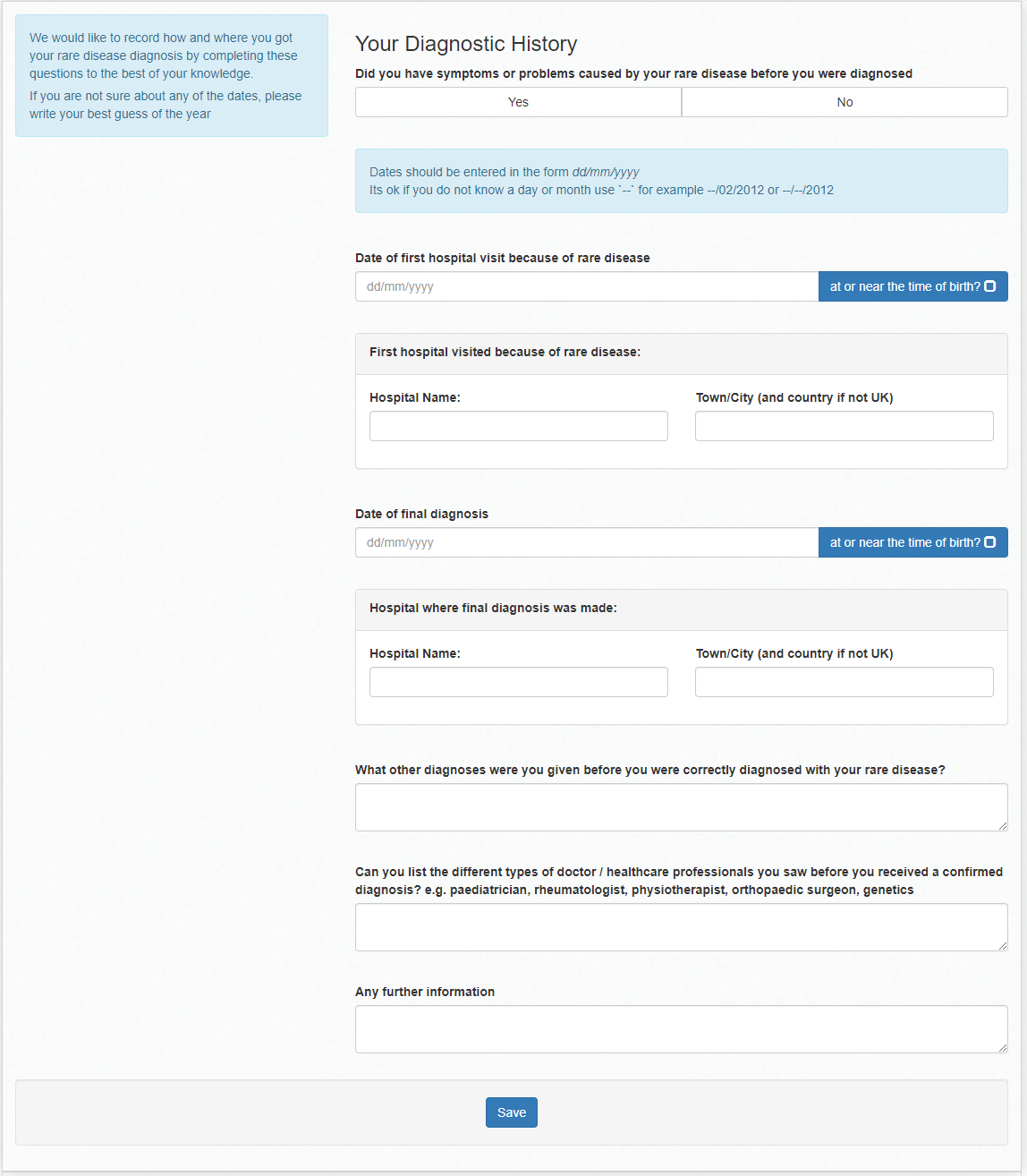


**Supplementary Table 1: Univariate and multivariate determinants for time from potential symptom onset to final diagnosis**

|  | **Univariate** | | |  | **Multivariate** | | |
| --- | --- | --- | --- | --- | --- | --- | --- |
| **Characteristic** | **Beta** | **95% CI^1^** | **p** |  | **Beta** | **95% CI^1^** | **p** |
| **Current age** | 0.46 | 0.22, 0.69 | <0.001 |  | 0.65 | 0.44, 0.87 | *<0.001**** |
| **Age at first symptom**  **onset** | -0.27 | -0.57, 0.03 | 0.081 |  | -0.75 | -1.1, -0.43 | *<0.001**** |
| **Sex (Female reference)** |  |  |  |  |  |  |  |
| Male | 2.0 | -7.3, 11 | 0.7 |  | 1.3 | -9.5, 7.5 | 0.8 |
| **Year of final diagnosis groups** |  |  |  |  |  |  |  |
| Before 2010 | — | — |  |  | — | — |  |
| After 2010 | 4.6 | -3.8, 13 | 0.3 |  | 18 | 11, 26 | *<0.001**** |
| **Other diagnosis given** |  |  |  |  |  |  |  |
| Correct | — | — |  |  | — | — |  |
| Incorrect | 0.47 | -8.1, 9.0 | >0.900 |  | 1.6 | -5.2, 8.5 | 0.600 |
| **See by orthopedic surgeon** |  |  |  |  |  |  |  |
| No | — | — |  |  |  |  |  |
| Yes | -3.9 | -9.9, 2.0 | 0.2 |  | 1.8 | -5.0, 8.6 | 0.6 |
| **Type of FD** |  |  |  |  |  |  |  |
| Monostotic | — | — |  |  | — | — |  |
| Cranio-facial | 1.9 | -11, 15 | 0.8 |  | -0.66 | -10, 9.2 | 0.9 |
| McCune Albright  Syndrome | -2.1 | -15, 11 | 0.7 |  | - 3.9 | -14, 6.5 | 0.5 |
| Polyostotic | 0.82 | -2.6, 28 | 0.10 |  | - 2.1 | -12, 7.8 | 0.7 |
| Not known | 13 | -12, 14 | 0.9 |  | 2.5 | -8.5, 14 | 0.6 |
| **First symptom descriptions** |  |  |  |  |  |  |  |
| Non pain | — | — |  |  | — | — |  |
| Pain | -3.1 | -12, 5.5 | 0.500 |  | -1.2 | -7.3, 9.8 | 0.8 |
| ***^1^*** *CI = Confidence Interval* | | | | | | | |

**Supplementary Table 2: Univariate and multivariate determinants for time from potential symptom onset to final diagnosis for those who were 18 and over when symptoms started**

|  | **Univariate** | | |  | | **Multivariate** | | | |
| --- | --- | --- | --- | --- | --- | --- | --- | --- | --- |
| **Characteristic** | **Beta** | **95% CI^1^** | **p** | |  | | **Beta** | **95% CI^1^** | **p** |
| **Current age** | 0.23 | -0.21, 0.67 | 0.3 | |  | | 0.57 | 0.22 0.92 | *0.006**** |
| **Age at first symptom**  **onset** | -0.43 | -0.90, 0.03 | 0.063 | |  | | -1.1 | -1.5, -0.73 | *<0.001* |
| **Sex (Female reference)** |  |  |  | |  | |  |  |  |
| Male | -2.6 | -14, 8.8 | 0.6 | |  | | -9.1 | -17, -1.6 | *0.024* |
| **Year of final diagnosis groups** |  |  |  | |  | |  |  |  |
| Before 2010 | — | — |  | |  | | — | — |  |
| After 2010 | 0.16 | -11, 12 | >0.9 | |  | | 9.6 | 3.1, 16 | *0.024* |
| **Seen by orthopedic** **surgeon** |  |  |  | |  | |  |  |  |
| No | — | — |  | |  | | — | — |  |
| Yes | 7.4 | -1.9, 17 | 0.11 | |  | | 5.5 | -0.87, 12 | 0.080 |
| **Other diagnosis given** |  |  |  | |  | |  |  |  |
| Correct | — | — |  | |  | | — | — |  |
| Incorrect | 2.9 | -7.2, 13 | 0.5 | |  | | 5.9 | -0.83, 13 | 0.077 |
| **Type of FD** |  |  |  | |  | |  |  |  |
| Monostotic | — | — |  | |  | | — | — |  |
| Cranio-facial | 3.3 | -10, 17 | 0.6 | |  | | 0.55 | -7.6, 8.7 | 0.9 |
| McCune Albright  Syndrome | 14 | -11, 38 | 0.3 | |  | | 0.13 | -14, 15 | >0.9 |
| Polyostotic | 6.7 | -13, 25 | 0.5 | |  | | 3.0 | -4.8, 11 | 0.4 |
| Not known | 6.0 | -8.1, 22 | 0.3 | |  | | 0.01 | -9.8, 11 | >0.9 |
| **First symptom descriptions** |  |  |  | |  | |  |  |  |
| Non pain | — | — |  | |  | | — | — |  |
| Pain | -3.1 | -12, 5.5 | 0.500 | |  | | 8.7 | -2.7, 20 | 0.12 |
| ***^1^*** *CI = Confidence Interval* | | | | | | | | | |
